# Dietary intake of choline and phosphatidylcholine and risk of type 2 diabetes in men: The Kuopio Ischaemic Heart Disease Risk Factor Study

**DOI:** 10.1007/s00394-020-02223-2

**Published:** 2020-03-20

**Authors:** Jyrki K. Virtanen, Tomi-Pekka Tuomainen, Sari Voutilainen

**Affiliations:** Institute of Public Health and Clinical Nutrition, University of Eastern Finland, Kuopio, Finland

**Keywords:** Choline, Phosphatidylcholine, Diet, Type 2 diabetes, Population study, Prospective study

## Abstract

**Purpose:**

To investigate associations of total dietary choline intake and its major dietary form, phosphatidylcholine, with type 2 diabetes risk.

**Methods:**

We included 2332 men aged 42–60 years at baseline in 1984–1989 from the Kuopio Ischaemic Heart Disease Risk Factor Study in eastern Finland. Dietary intakes were assessed with 4-d food recording at baseline. Type 2 diabetes diagnosis was based on self-administered questionnaires, fasting and 2-h oral glucose tolerance test blood glucose measurements, or by record linkage to national health registries. Multivariable-adjusted Cox proportional hazards regression models were used for statistical analysis.

**Results:**

During the mean 19.3-year follow-up, 432 men had type 2 diabetes diagnosis. After multivariable adjustments, those in the highest vs. lowest choline intake quartile had 25% (95% CI 2–43%) lower relative risk *(P* trend across quartiles = 0.02) and those in the highest vs. lowest phosphatidylcholine quartile had 41% (95% CI 22–55%) lower relative risk (*P *trend < 0.001) of type 2 diabetes.

**Conclusions:**

Higher choline intake, especially phosphatidylcholine, was associated with lower type 2 diabetes risk among men.

**Electronic supplementary material:**

The online version of this article (10.1007/s00394-020-02223-2) contains supplementary material, which is available to authorized users.

## Introduction

Choline is an essential nutrient for humans [1]. In diet, it exists as free choline and as choline esters, of which phosphatidylcholine is the major dietary source of choline. Although choline is found in most foods, animal products, such as meat and dairy and especially eggs and liver, are rich choline sources. Choline has an important role in human metabolism by acting as a methyl donor, a precursor for neurotransmitter acetylcholine and a component of cell membranes [1]. However, choline is also a major dietary precursor for gut microbiome-derived trimethylamine, which is converted in liver to trimethylamine N-oxide (TMAO) [2]. Elevated blood TMAO concentration has been implicated as a risk factor for atherosclerosis, cardiovascular disease, kidney disease and mortality, and also for type 2 diabetes (T2D) [2, 3].

Because choline is a major precursor for TMAO, high choline intake could have an adverse impact on T2D risk. However, few studies have investigated this association and the findings are mixed. In one cross-sectional study, a higher dietary choline intake was associated with lower insulin resistance [4]. An opposite finding was obtained from a large prospective study, which found a higher risk of incident T2D with a higher dietary phosphatidylcholine intake [5].

Because of the conflicting and scarce evidence of the role of choline in the development of T2D, we investigated the associations of intake of choline and phosphatidylcholine with risk of incident T2D among middle-aged men from eastern Finland.

## Materials and methods

The Kuopio Ischaemic Heart Disease Risk Factor Study (KIHD) is a prospective cohort study in an age-stratified random sample of men from eastern Finland [6]. The baseline examinations were carried out in 1984–1989 for 2682 men aged 42–60 years and were followed by three subsequent examination rounds (Supplemental Fig. 1). From the analyses, we excluded subjects with baseline T2D (*n* = 167), impaired fasting glucose (*n* = 127, defined as fasting plasma glucose 6.1–6.9 mmol/L) or unknown diabetes status (*n* = 38), or those without data on dietary intakes (*n* = 18), which left 2332 men. There was no missing data on blood glucose measurements.

**Fig. 1:**
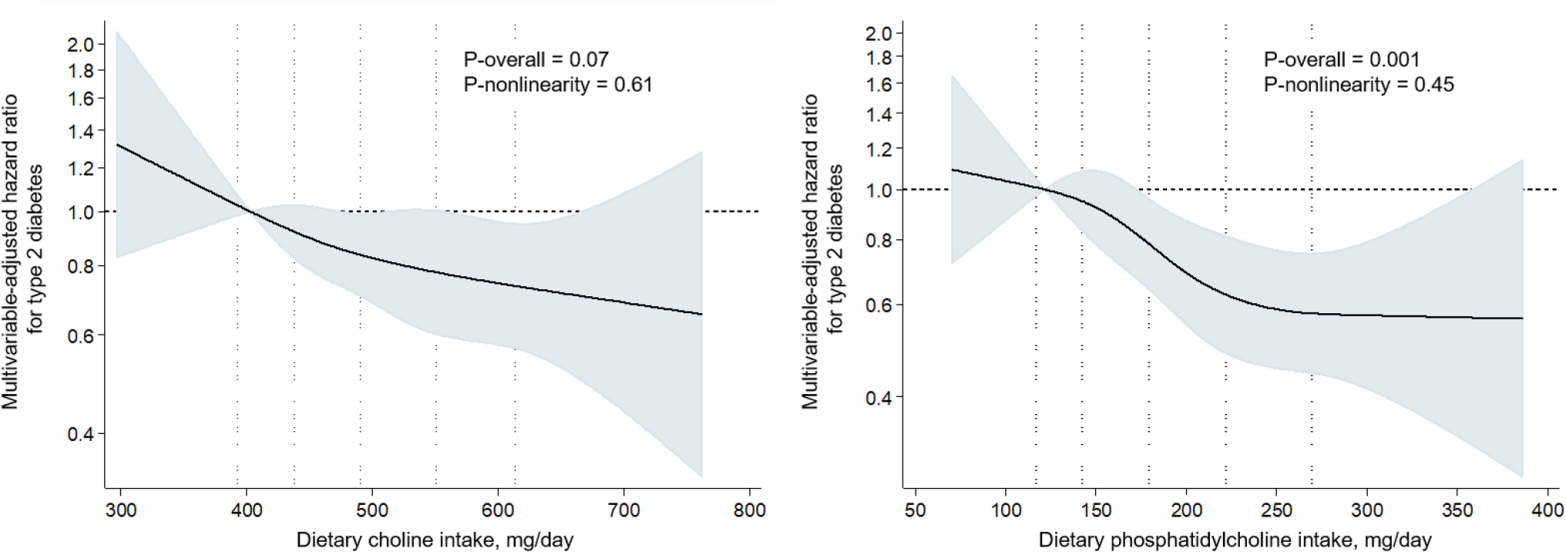
Multivariable-adjusted hazard ratios of dietary choline and phosphatidylcholine intake with risk of type 2 diabetes among 2332 men, evaluated by restricted cubic splines from Cox proportional hazards models. The models are adjusted for age, examination year, smoking (never smoker, previous smoker, current smoker < 20 cigarettes/day and current smoker ≥ 20 cigarettes/day), body mass index (kg/m^2^), leisure-time physical activity (kcal/day), family history of type 2 diabetes (yes/no), and intakes of energy (kcal/day), alcohol (g/week), polyunsaturated fatty acids (percent of energy), and fiber (g/day). The solid lines represent the central risk estimates and the shades are the 95% confidence interval, relative to the reference level (12.5th percentile). The dotted vertical lines correspond to 10th, 25th, 50th, 75th and 90th percentile of the dietary intakes

Detailed descriptions of the determination of serum lipids and lipoproteins, assessment of medical history and medications, family history of diseases, smoking, alcohol consumption, and blood pressure have been published [6]. Physical activity was evaluated based on the 12-month leisure-time physical activity questionnaire and expressed as kcal/day. The most common leisure-time physical activities were recorded, including the average duration, intensity, and frequency of each activity. Information on education and annual income was obtained from a self-administered questionnaire. The family history of diabetes was defined as “yes”, if a first-degree relative had diabetes history. Hypertension diagnosis was defined as systolic/diastolic blood pressure > 140/90 mmHg or use of hypertension medication. An immunometric assay (Immulite High Sensitivity C-reactive Protein Assay, DPC, Los Angeles, USA) was used to measure serum high-sensitivity CRP.

Dietary intakes were assessed with instructed 4-day food recording at baseline. Nutrient intakes were estimated using the NUTRICA® 2.5 software (Social Insurance Institution, Turku, Finland). The databank of the software is mainly based on Finnish values of nutrient composition of foods. Because there is no information on choline and phosphatidylcholine values in Finnish foods, these values are based on the USDA database (https://www.ars.usda.gov/SP2UserFiles/Place/80400525/Data/Choline/Choln02.pdf). All nutrients were adjusted for total energy intake using the residual method.

T2D was defined as a self-reported physician-set diagnosis of T2D and/or fasting plasma glucose ≥ 7.0 mmol/L or 2-h oral glucose tolerance test plasma glucose ≥ 11.1 mmol/L at re-examination rounds 4, 11 and 20 years after the baseline, or by record linkage to the national hospital discharge registry and to the Social Insurance Institution of Finland register for reimbursement of medicine expenses used for T2D for the entire study period until the end of follow-up in Dec 31, 2010. The 2-h oral glucose tolerance test was not done at the study baseline. We did not have data on HbA1c. The participants contributed follow-up time until T2D diagnosis at a re-examination round or until the date of T2D diagnosis based on hospital discharge registry or the register for reimbursement of medicine expenses during the follow-up, or death, whichever came first.

The univariate associations of choline and phosphatidylcholine intake quartiles with baseline characteristics were explored by means and linear regression for continuous variables and *χ*^2^ tests for bivariate relationships. Hazard ratios (HR) for T2D incidence in quartiles were obtained from the Cox proportional hazards regression models, with the lowest category as the reference. The validity of the proportional hazards assumption was evaluated using Schoenfeld residuals and the assumptions were met. Absolute risk reduction (ARR) was calculated by multiplying the absolute risk (AR) in the reference group by the multivariable-adjusted HR reduction in the comparison group. Because exact time-to-event data were not available for those T2D cases that were diagnosed at the re-examination rounds, in the sensitivity analyses we also investigated the associations using logistic regression. We selected the potential confounders (listed under Table 1) for the analyses based on established risk factors for T2D, previous publications of dietary choline intake with T2D risk, or on associations with exposures or outcomes in the KIHD cohort. Missing values (< 2.5%) in covariates were replaced with the cohort mean. Linear trends across quartiles of choline and phosphatidylcholine intake were assessed by assigning the median intake value for each quartile and then entering that as a continuous variable in the statistical models (Table 1, supplemental tables 2, 3). Potential nonlinear associations were assessed semiparametrically using restricted cubic splines. Statistical significance of the linear interactions with BMI and age medians on a multiplicative scale was assessed by likelihood ratio tests using a cross-product term with choline and phosphatidylcholine as continuous variables. All *P* values were two-tailed (*α* = 0.05). Data were analyzed using SPSS 23.0 for Windows (Armonk, NY: IBM Corp.) and Stata 14.1 (Stata Corp., College Station, Texas; for spline analysis).

**Table 1:** Hazard ratios (95% confidence intervals) of incident type 2 diabetes according to quartiles of dietary choline and phosphatidylcholine intake

	Intake quartile				*P* trend
	1 (*n* = 583)	2 (*n* = 583)	3 (*n* = 583)	4 (*n* = 583)	
Choline (mg/day)	< 373	373–423	424–482	> 482	
Number of events	117	113	101	101	
Incidence rate/1000 PY	10.6	10.1	8.9	8.9	
Model 1	1	0.92 (0.71–1.19)	0.80 (0.61–1.04)	0.84 (0.65–1.10)	0.15
Model 2	1	0.94 (0.73–1.22)	0.78 (0.62–1.02)	0.75 (0.57–0.98)	0.02
Model 3	1	0.89 (0.68–1.17)	0.69 (0.51–0.94)	0.65 (0.43–0.96)	0.02
Model 4	1	0.91 (0.69–1.20)	0.71 (0.51–0.98)	0.68 (0.43–1.06)	0.06
Phosphatidylcholine (mg/day)	< 142	142–179	180–222	> 222	
Number of events	125	109	101	97	
Incidence rate/1000 PY	11.9	9.7	8.8	8.4	
Model 1	1	0.79 (0.61–1.02)	0.70 (0.54–0.91)	0.68 (0.52–0.88)	0.004
Model 2	1	0.78 (0.60–1.01)	0.72 (0.55–0.94)	0.59 (0.45–0.78)	< 0.001
Model 3	1	0.76 (0.57–1.00)	0.69 (0.50–0.96)	0.56 (0.36–0.89)	0.02
Model 4	1	0.76 (0.57–1.01)	0.70 (0.50–0.99)	0.57 (0.34–0.94)	0.03

Model 1 is adjusted for age, examination year and energy intake (kcal/day)

Model 2 is adjusted for Model 1 plus smoking (never smoker, previous smoker, current smoker < 20 cigarettes/day and current smoker ≥ 20 cigarettes/day), body mass index (kg/m^2^), leisure-time physical activity (kcal/day), family history of type 2 diabetes (yes/no), and intakes of alcohol (g/week), polyunsaturated fatty acids (percent of energy), and fiber (g/day)

Model 3 adjusted for Model 2 and the major dietary sources of choline (dairy, meat and eggs) and phosphatidylcholine (eggs and meat)

Model 4 adjusted for Model 3 and energy-adjusted intake of nutrients involved in the choline metabolism, vitamin B_12_ and folate

*PY* person–years

## Results

The mean energy-adjusted choline intake was 430.9 mg/day (SD 89.8). Phosphatidylcholine accounted for 43.6% (187.9 mg/day, SD 64.6) of the total choline intake. Dairy, meat and eggs were the major contributors to choline intake, whereas eggs and meat were the major phosphatidylcholine sources (Supplemental Table 1).

Supplemental Tables 2 and 3 show the baseline characteristics according to choline and phosphatidylcholine intakes. Both were associated with higher intake of eggs, meat, fruits, berries and vegetables, and fish and lower intake of grains and fats. In contrast, choline intake was associated with a higher and phosphatidylcholine intake with a lower dairy intake. Regarding nutrients, both were associated with a higher cholesterol intake but with lower intake of saturated fatty acids and higher intake of unsaturated fatty acids, and with lower intake of carbohydrates and lower glycemic load. Phosphatidylcholine intake was also associated with lower fiber intake. Among the other factors, both were associated with lower prevalence of coronary heart disease and those with higher phosphatidylcholine intake were also more likely to be younger, have a higher income and education and were less likely to have hypertension.

During the mean follow-up of 19.3 years (SD 6.6), 432 men (18.5%) had T2D diagnosis. After multivariable adjustments (Model 2), those in the highest vs. lowest choline intake quartile had 25% (95% CI 2–43%) lower relative risk (*P* trend across quartiles = 0.02) and those in the highest vs. lowest phosphatidylcholine intake quartile had 41% (95% CI 22–55%) lower relative risk (*P* trend < 0.001) (Table 1, Supplemental Fig. 2). The absolute risk was 5.0% lower in the highest vs the lowest choline intake quartile (absolute risk in the lowest quartile 20.1%) and 8.6% lower in the highest vs. the lowest phosphatidylcholine intake quartile (absolute risk in the lowest quartile 21.4%). Restricted cubic splines analysis showed relatively linear associations of choline and phosphatidylcholine intakes with lower risk of T2D (for overall associations *P* = 0.07 and *P* = 0.001, respectively) and no evidence for nonlinearity (*P* = 0.61 and *P* = 0.45, respectively) (Fig. 1). Each 100 mg/day higher choline intake was associated with the HR = 0.91 (95% CI 0.81–1.01) and each 100 mg/day higher phosphatidylcholine intake with the HR = 0.77 (95% CI 0.65–0.90). Further adjustments for the major dietary sources of choline or phosphatidylcholine (Model 3) or for nutrients involved in the choline metabolism (Model 4) had little impact on the associations (Table 1). Additional adjustment for the intake of other potentially confounding dietary factors that were higher (fish, and fruits, berries and vegetables) or lower (saturated fatty acids) among those with higher choline and phosphatidylcholine intakes (Supplemental Table 1) had no appreciable impact on the associations (data not shown). Excluding subjects with history of coronary heart disease at baseline (*n* = 545) did not appreciably change the association with choline intake (HR in the highest vs. lowest quartile 0.77, 95% CI 0.57–1.04, *P* trend = 0.07, Model 2), but modestly attenuated the association with phosphatidylcholine intake (extreme quartile HR = 0.71, 95% CI 0.52–0.97, *P* trend = 0.04). The associations were similar, if we excluded the T2D events that occurred during the first 5 years of follow-up (*n* = 35) or included in the analyses only men with complete data on all covariates (*n* = 2255, 418 T2D events, data not shown). Age or BMI did not modify the associations (*P* interactions > 0.05). Finally, the associations were also similar if we used logistic regression instead of Cox proportional hazards regression. For example, the extreme quartile odds ratio for choline intake was 0.73 (95% CI 0.54–1.00, *P* trend = 0.02) and for phosphatidylcholine intake 0.60 (95% CI 0.44–0.81, *P* trend = 0.002) (Model 2, other data not shown).

## Discussion

The main finding of this prospective cohort study among middle-aged and older eastern Finnish men was that choline and phosphatidylcholine intakes were associated with a lower risk of developing T2D during the 19-year follow-up.

Our findings are supported by a cross-sectional study that found a beneficial association between dietary choline intake and glucose metabolism indices among men and women with the mean age of 43.4 years and the mean choline intake of 305 mg/day [4]. However, our results are in contrast with a large prospective study from Harvard that pooled results from the Nurses’ Health Study I and II and the Health Professionals Follow-up Study and found that higher phosphatidylcholine intake was associated with increased risk of T2D and suggested conversion to TMAO as a possible explanation for the association [5]. Unfortunately, this paper did not provide detailed information on the study populations for comparison with the current study. There is no apparent explanation for these divergent findings, because the Harvard study and our study used the same database for the choline content of foods. However, one option could be differences in gut microbiota, because not all strains of bacteria can convert choline to trimethylamine [7]. Possibly because of this, there is substantial interindividual, and perhaps also interpopulation, variation in the circulating TMAO concentrations in response to choline intake [7]. Unfortunately, microbiome data are rarely available in large cohort studies, including our study. Another explanation could be the differences in the dietary sources of choline. However, choline is primarily found in 3–4 major food groups that are commonly consumed across different populations and adjusting for the major sources had little impact on the associations in our study or in the other prospective study [5]. Of note, intake of eggs, the major phosphatidylcholine source did not correlate with circulating TMAO concentrations in the KIHD cohort, but was associated with a lower T2D risk [8]. It is noteworthy that there may also be interpopulation differences in the relationship of circulating TMAO with T2D risk. A study from Norway, a northern European country like Finland, did not find evidence for an association between circulating TMAO and risk of incident T2D [9], whereas a study from China found circulating TMAO concentrations to associate with increased T2D risk [3]. In contrast, a study from Spain found a strong inverse association between baseline TMAO and risk of incident T2D [10]. Although these inconsistent findings may be due to differences in technical assays used for TMAO measurement, they may also suggest possible ethnic differences in the TMAO–T2D relationship. Overall, because the role of TMAO in the development of T2D is still unclear and there is significant variation in the response of circulation TMAO concentrations to choline intake, it is currently difficult to conclude whether TMAO has a role in the dietary choline–T2D relationship. Therefore, future research is needed to elucidate the role of other choline-related metabolites and also the other numerous functions that choline has in the body, besides being a precursor for TMAO [1].

Strengths of our study include detailed information on T2D events and on potential confounders, large number of events and no loss to follow-up. Limitations include the single assessment of dietary intakes at baseline. Because dietary habits may change over time, this can introduce misclassification and random error and thus attenuate the associations during a long follow-up. There is no information on choline content of foods in Finland, so we needed to rely on the analyses conducted in the USA, which may not completely represent the choline content in Finnish foods. A major limitation is that we did not have information on circulating TMAO concentrations. The T2D diagnoses based on data from the national registries have not been validated by us and we did not have information on the actual date of the diagnosis for the diagnoses that were based solely on the study visit data. However, inaccuracies in the T2D diagnosis would most likely rather attenuate than strengthen the associations. Because the study population included only Caucasian men from eastern Finland, the findings may not be generalizable to other populations.

In conclusion, higher choline intake and especially phosphatidylcholine intake was associated with lower risk of T2D in the eastern Finnish male population. Because of the conflicting and limited research data, future studies are needed to elucidate the role of choline intake in glucose homeostasis, preferably taking into account also the impact of microbiota.

## Electronic supplementary material

Below is the link to the electronic supplementary material.

Supplementary file1 (DOCX 222 kb)
