# Supplementary material for: Dietary intake of choline and phosphatidylcholine and risk of type 2 diabetes in men: The Kuopio Ischaemic Heart Disease Risk Factor Study
Source: Eur J Nutr. 2020 Mar 20;59(8):3857–61. doi: 10.1007/s00394-020-02223-2 (PMC7669791; doi:10.1007/s00394-020-02223-2)
Supplement: Supplementary file 1 — Supplementary file1 (DOCX 222 kb) [file 394_2020_2223_MOESM1_ESM.docx]

**European Journal of Nutrition**

SUPPLEMENTAL DATA

**Dietary intake of choline and phosphatidylcholine and risk of type 2 diabetes in men: The Kuopio Ischaemic Heart Disease Risk Factor Study**

Jyrki K Virtanen^1^, Tomi-Pekka Tuomainen^1^, Sari Voutilainen^1^

^1^University of Eastern Finland, Institute of Public Health and Clinical Nutrition, Kuopio, Finland.

Corresponding author: Jyrki K Virtanen, University of Eastern Finland, Institute of Public Health and Clinical Nutrition, Kuopio, Finland. Phone: +358-40-3552957, E-mail: jyrki.virtanen@uef.fi.

**Supplemental Table 1** Dietary sources of choline and phosphatidylcholine

| Choline  (total intake 430.9 mg/d) | |  | Phosphatidylcholine  (total intake 187.9 mg/d) | |
| --- | --- | --- | --- | --- |
| Source | % of total intake |  | Source | % of total intake |
| Dairy | 26.5 |  | Eggs | 39.3 |
| Meat | 24.0 |  | Meat | 36.4 |
| Eggs | 18.6 |  | Fruits, berries, roots and other vegetables | 6.6 |
| Grains | 11.0 |  | Fish | 5.2 |
| Fruits, berries, roots and other vegetables | 10.9 |  | Grains | 4.8 |
| Fish | 6.6 |  | Dairy | 4.6 |
| Butter and margarines | 1.8 |  | Butter and margarines | 2.8 |
| Other | 0.6 |  | Other | 0.3 |

Meat intake includes processed and unprocessed red meat, white meat and offal. There were no users of processed white meat.

**Supplemental Table 2** Baseline characteristics according to quartiles of dietary choline intake: The Kuopio Ischaemic Heart Disease Risk Factor Study (n = 2,332)

|  | Choline intake (mg/d) | | | |
| --- | --- | --- | --- | --- |
|  | 1 (<373) | 2 (373-423) | 3 (424-481) | 4 (>481) |
| Number of subjects | 583 | 583 | 583 | 583 |
| *Dietary intakes* |  |  |  |  |
| Energy (kcal/d) | 2515 ± 667 | 2356 ± 586 | 2404 ± 576 | 2554 ± 622 |
| Phosphatidylcholine (mg/d) | 131 ± 32 | 167 ± 34 | 195 ± 38 | 258 ± 66* |
| Choline (mg/d) | 329 ± 42 | 397 ± 15 | 450 ± 16 | 547 ± 67* |
| Eggs (g/d) | 18 ± 13 | 25 ± 16 | 32 ± 19 | 53 ± 33* |
| Meat (g/d) | 135 ± 71 | 145 ± 73 | 165 ± 77 | 186 ± 88* |
| Unprocessed red meat (g/d) | 54 ± 38 | 66 ± 44 | 75 ± 48 | 78 ± 55* |
| Processed red meat (g/d) | 69 ± 60 | 64 ± 56 | 68 ± 59 | 76 ± 63* |
| Offal (g/d) | 3 ± 9 | 2 ± 7 | 5 ± 11 | 10 ± 19* |
| White meat (g/d) | 6 ± 20 | 9 ± 24 | 11 ± 29 | 13 ± 33* |
| Dairy (g/d) | 615 ± 334 | 659 ± 330 | 736 ± 345 | 845 ± 387* |
| Fermented dairy (g/d) | 124 ± 147 | 181 ± 203 | 196 ± 212 | 247 ± 273* |
| Non-fermented dairy (g/d) | 491 ± 321 | 479 ± 312 | 540 ± 318 | 597 ± 361* |
| Grains (g/d) | 274 ± 100 | 251 ± 88 | 244 ± 88 | 251 ± 95* |
| Whole grains (g/d) | 170 ± 85 | 156 ± 71 | 154 ± 72 | 159 ± 75* |
| Fish (g/d) | 35 ± 41 | 40 ± 45 | 47 ± 49 | 62 ± 72* |
| Fruits, berries, roots and other vegetables (g/d) | 370 ± 162 | 408 ± 174 | 428 ± 175 | 452 ± 180* |
| Fats (g/d) | 62 ± 28 | 54 ± 22 | 53 ± 22 | 55 ± 24* |
| Butter and butter containing spreads (g/d) | 42 ± 33 | 35 ± 25 | 34 ± 25 | 35 ± 26* |
| Vegetable margarines (g/d) | 18 ± 19 | 17 ± 16 | 17 ± 15 | 18 ± 17 |
| Vegetable oils (g/d) | 2 ± 4 | 2 ± 3 | 2 ± 4 | 2 ± 3 |
| Coffee (mL/d) | 588 ± 303 | 554 ± 287 | 547 ± 288 | 579 ± 301 |
| Protein (E%) | 13.8 ± 1.9 | 15.2 ± 1.9 | 16.3 ± 2.1 | 17.3 ± 2.4* |
| Saturated fatty acids (E%) | 18.9 ± 4.3 | 18.2 ± 4.0 | 17.8 ± 3.9 | 17.8 ± 3.9* |
| Polyunsaturated fatty acids (E%) | 4.3 ± 1.5 | 4.4 ± 1.4 | 4.6 ± 1.4 | 4.6 ± 1.3* |
| Monounsaturated fatty acids (E%) | 11.5 ± 2.2 | 11.6 ± 2.1 | 11.7 ± 2.2 | 11.9 ± 2.1* |
| Trans fatty acids (E%) | 1.1 ± 0.4 | 1.1 ± 0.3 | 1.0 ± 0.3 | 1.0 ± 0.4* |
| Cholesterol (mg/d) | 333 ± 78 | 371 ± 72 | 405 ± 85 | 495 ± 121* |
| Carbohydrates (E%) | 43.9 ± 6.6 | 41.5 ± 6.2 | 44.3 ± 6.7 | 41.5 ± 6.2* |
| Glycemic load | 179 ± 56 | 165 ± 51 | 163 ± 49 | 164 ± 50* |
| Glycemic index | 66 ± 6 | 65 ± 5 | 64 ± 5 | 63 ± 5* |
| Fiber (g/d) | 24.7 ± 8.0 | 25.6 ± 6.6 | 25.2 ± 6.7 | 24.9 ± 7.4 |
| Age (y) | 52.8 ± 5.2 | 53.3 ± 5.1 | 53.2 ± 5.1 | 52.6 ± 5.2 |
| Body mass index (kg/m^2^) | 26.4 ± 3.3 | 26.4 ± 3.2 | 26.6 ± 3.3 | 27.0 ± 3.6* |
| Leisure-time physical activity (kcal/d) | 136 ± 167 | 145 ± 181 | 139 ± 163 | 141 ± 185 |
| Income (euro) | 12282 ± 7939 | 13894 ± 9192 | 14076 ± 9190 | 12944 ± 9331 |
| Education (y) | 8.6 ± 3.5 | 8.7 ± 3.4 | 8.8 ± 3.4 | 8.6 ± 3.4 |
| Alcohol intake (g/wk) | 86 ± 191 | 65 ± 96 | 64 ± 110 | 74 ± 108 |
| Serum LDL cholesterol (mmol/L) | 4.06 ± 1.08 | 4.08 ± 1.01 | 4.01 ± 1.02 | 4.05 ± 0.98 |
| Serum HDL cholesterol (mmol/L) | 1.30 ± 0.31 | 1.30 ± 0.30 | 1.29 ± 0.28 | 1.32 ± 0.32 |
| Serum triglycerides (mmol/L) | 1.28 ± 0.76 | 1.31 ± 0.71 | 1.22 ± 0.66 | 1.26 ± 0.85 |
| Blood glucose, mmol/L | 4.52 ± 0.40 | 4.51 ± 0.38 | 4.50 ± 0.40 | 4.55 ± 0.41 |
| Serum insulin, mU/L | 10.27 ± 5.97 | 10.87 ± 6.02 | 10.65 ± 5.34 | 11.60 ± 7.06* |
| Serum C-reactive protein, | 2.55 ± 5.87 | 2.13 ± 2.79 | 2.21 ± 3.23 | 2.36 ± 3.47 |
| Current smoker (%) | 36 | 30 | 30 | 33 |
| Hypertension (%) | 60 | 57 | 59 | 57 |
| Coronary heart disease (%) | 27 | 23 | 23 | 20* |
| Stroke (%) | 2 | 3 | 2 | 2 |
| Family history of type 2 diabetes (%) | 27 | 26 | 26 | 28 |

Values are means ± SD or percentages.

**P* for trend across quartiles <0.05.

E% = percent of energy.

**Supplemental Table 3** Baseline characteristics according to quartiles of dietary phosphatidylcholine intake: The Kuopio Ischaemic Heart Disease Risk Factor Study (n = 2,332)

|  | Phosphatidylcholine intake (mg/d) | | | |
| --- | --- | --- | --- | --- |
|  | 1 (<142) | 2 (142-179) | 3 (180-222) | 4 (>222) |
| Number of subjects | 583 | 583 | 583 | 583 |
| *Dietary intakes* |  |  |  |  |
| Energy (kcal/d) | 2522 ± 673 | 2385 ± 596 | 2403 ± 582 | 2519 ± 608 |
| Phosphatidylcholine (mg/d) | 117 ± 21 | 161 ± 11 | 199 ± 12 | 274 ± 53* |
| Choline (mg/d) | 350 ± 60 | 404 ± 54 | 442 ± 49 | 527 ± 83* |
| Eggs (g/d) | 14 ± 9 | 22 ± 12 | 33 ± 15 | 60 ± 30* |
| Meat (g/d) | 123 ± 68 | 149 ± 72 | 164 ± 70 | 195 ± 90* |
| Unprocessed red meat (g/d) | 48 ± 34 | 65 ± 42 | 75 ± 48 | 86 ± 56* |
| Processed red meat (g/d) | 64 ± 56 | 67 ± 60 | 67 ± 55 | 78 ± 66* |
| Offal (g/d) | 3 ± 9 | 4 ± 10 | 5 ± 12 | 8 ± 17* |
| White meat (g/d) | 4 ± 16 | 9 ± 26 | 11 ± 28 | 14 ± 35* |
| Dairy (g/d) | 815 ± 373 | 710 ± 359 | 660 ± 321 | 669 ± 365* |
| Fermented dairy (g/d) | 198 ± 235 | 199 ± 234 | 174 ± 181 | 177 ± 216* |
| Non-fermented dairy (g/d) | 617 ± 353 | 511 ± 320 | 486 ± 307 | 492 ± 329* |
| Grains (g/d) | 272 ± 102 | 256 ± 89 | 248 ± 88 | 246 ± 92* |
| Whole grains (g/d) | 177 ± 86 | 161 ± 70 | 153 ± 73 | 148 ± 71* |
| Fish (g/d) | 41 ± 45 | 44 ± 48 | 48 ± 56 | 50 ± 65* |
| Fruits, berries, roots and other vegetables (g/d) | 380 ± 169 | 409 ± 178 | 441 ± 173 | 427 ± 177* |
| Fats (g/d) | 61 ± 29 | 53 ± 21 | 54 ± 22 | 56 ± 23* |
| Butter and butter containing spreads (g/d) | 43 ± 34 | 34 ± 25 | 33 ± 25 | 34 ± 26* |
| Vegetable margarines (g/d) | 17 ± 18 | 17 ± 16 | 18 ± 16 | 19 ± 17* |
| Vegetable oils (g/d) | 2 ± 3 | 2 ± 4 | 3 ± 4 | 2 ±4 |
| Coffee (mL/d) | 594 ± 321 | 551 ± 272 | 562 ± 279 | 561 ± 306 |
| Protein (E%) | 14.5 ± 2.1 | 15.4 ± 2.2 | 16.1 ± 2.3 | 16.7 ± 2.6* |
| Saturated fatty acids (E%) | 19.0 ± 6.3 | 18.0 ± 3.9 | 17.7 ± 3.7 | 18.1 ± 3.7* |
| Polyunsaturated fatty acids (E%) | 4.0 ± 1.4 | 4.4 ± 1.4 | 4.7 ± 1.4 | 4.8 ± 1.4* |
| Monounsaturated fatty acids (E%) | 11.0 ± 2.1 | 11.5 ± 2.1 | 11.7 ± 2.2 | 12.3 ± 2.1* |
| Trans fatty acids (E%) | 1.1 ± 0.4 | 1.0 ± 0.4 | 1.0 ± 0.4 | 1.0 ± 0.4* |
| Cholesterol (mg/d) | 322 ± 75 | 362 ± 68 | 405 ± 71 | 515 ± 107* |
| Carbohydrates (E%) | 44.3 ± 6.7 | 44.1 ± 6.1 | 43.0 ± 6.0 | 40.7 ± 6.1* |
| Glycemic load | 177 ± 56 | 167 ± 50 | 165 ± 50 | 162 ± 51* |
| Glycemic index | 65 ± 6 | 65 ± 6 | 65 ± 5 | 64 ± 5 |
| Fiber (g/d) | 25.6 ± 8.2 | 25.8 ± 7.0 | 25.2 ± 6.5 | 23.8 ± 7.0* |
| Age (y) | 53.6 ± 4.9 | 53.3 ± 5.1 | 53.0 ± 5.0 | 52.1 ± 5.4* |
| Body mass index (kg/m^2^) | 26.6 ± 3.5 | 26.6 ± 3.3 | 26.4 ± 3.1 | 26.8 ± 3.4 |
| Leisure-time physical activity (kcal/d) | 130 ± 169 | 136 ± 161 | 156 ± 205 | 138 ± 157 |
| Income (euro) | 11198 ± 7347 | 13124 ± 7944 | 15102 ± 10858 | 13778 ± 8861* |
| Education (y) | 7.9 ± 3.1 | 8.7 ± 3.5 | 9.1 ± 3.7 | 9.0 ± 3.5* |
| Alcohol intake (g/wk) | 74 ± 143 | 67 ± 153 | 68 ± 111 | 81 ± 116 |
| Serum LDL cholesterol (mmol/L) | 4.07 ± 1.08 | 4.13 ± 1.02 | 4.02 ± 1.00 | 3.97 ± 0.97* |
| Serum HDL cholesterol (mmol/L) | 1.30 ± 0.31 | 1.29 ± 0.31 | 1.31 ± 0.30 | 1.31 ± 0.30 |
| Serum triglycerides (mmol/L) | 1.32 ± 0.80 | 1.28 ± 0.70 | 1.20 ± 0.62 | 1.26 ± 0.85 |
| Blood glucose, mmol/L | 4.53 ± 0.40 | 4.53 ± 0.39 | 4.49 ± 0.40 | 4.55 ± 0.41 |
| Serum insulin, mU/L | 10.69 ±6.33 | 10.79 ± 6.09 | 10.50 ± 4.84 | 11.40 ± 7.07 |
| Serum C-reactive protein, | 2.58 ± 4.63 | 2.31 ± 4.71 | 2.02 ± 3.01 | 2.34 ± 3.44 |
| Current smoker (%) | 39 | 30 | 27 | 33 |
| Hypertension (%) | 62 | 61 | 54 | 56* |
| Coronary heart disease (%) | 30 | 24 | 20 | 20* |
| Stroke (%) | 2 | 3 | 2 | 2 |
| Family history of type 2 diabetes (%) | 25 | 28 | 26 | 29 |

Values are means ± SD or percentages.

**P* for trend across quartiles <0.05.

E% = percent of energy.


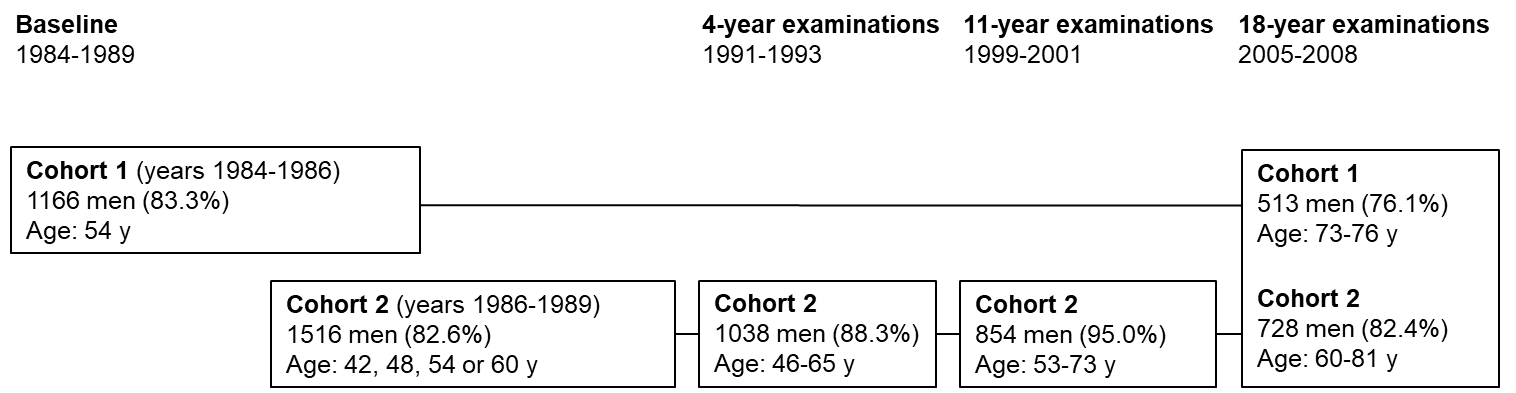


**Supplemental Figure 1.** The timeline of the Kuopio Ischaemic Heart Disease Risk Factor Study (KIHD). Percentages in brackets indicate the proportion of the eligible participants that participated in the study visits.


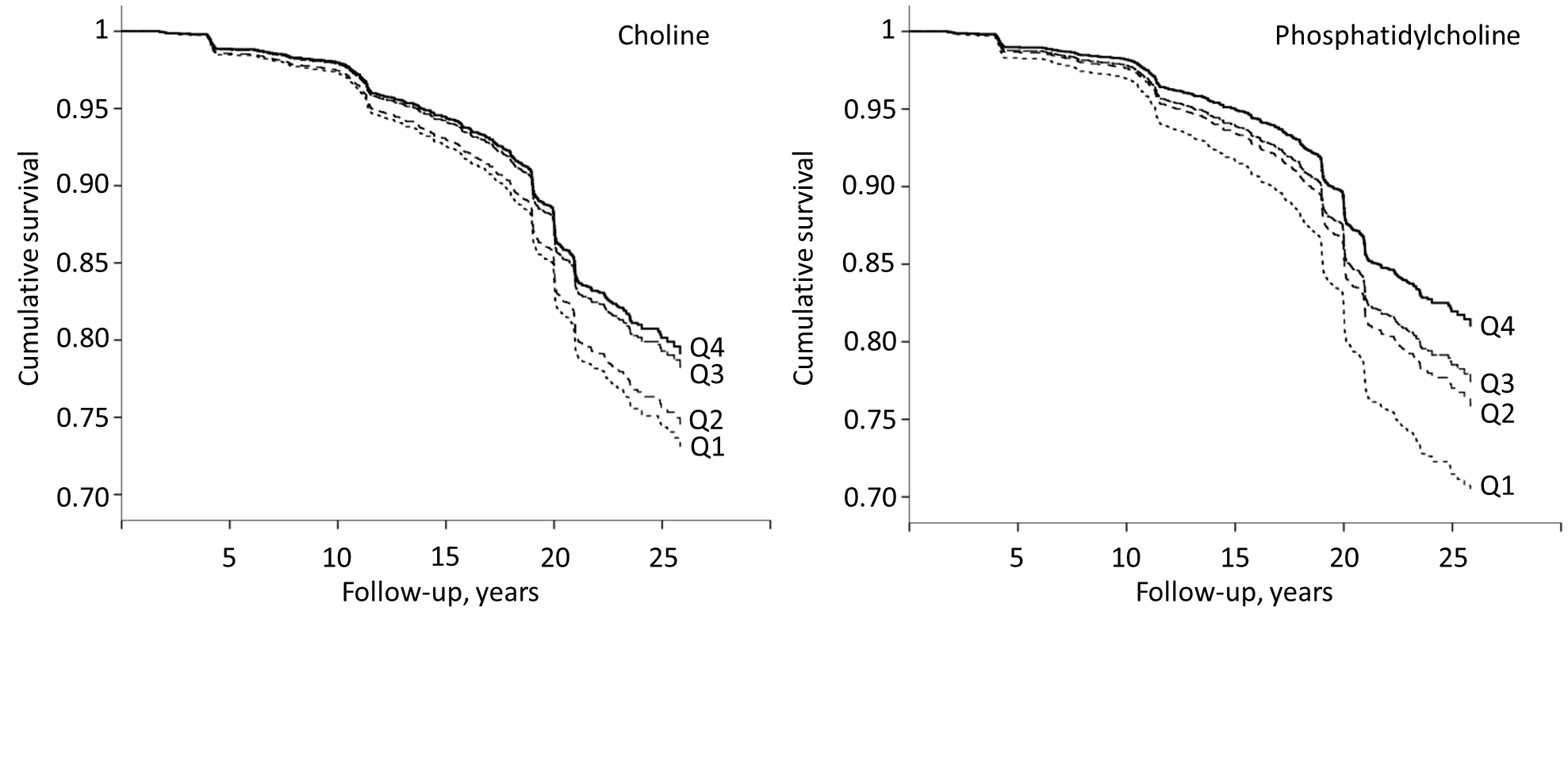
**Supplemental Figure 2.** Survival free of type 2 diabetes during the follow up of the Kuopio Ischaemic Heart Disease Risk Factor Study, according to the quartiles of baseline energy-adjusted dietary choline and phosphatidylcholine intakes in 2332 men. The model is adjusted for age, examination year, smoking (never smoker, previous smoker, current smoker <20 cigarettes/day and current smoker ≥20 cigarettes/day), body mass index (kg/m^2^), leisure-time physical activity (kcal/day), family history of type 2 diabetes (yes/no), and intakes of alcohol (g/day), energy (kcal/day), polyunsaturated fatty acids (percent of energy), and fiber (g/day). Q, quartile.
